# Young adult-onset, very slowly progressive cognitive decline with spastic paraparesis in Alzheimer’s disease with cotton wool plaques due to a novel presenilin1 G417S mutation

**DOI:** 10.1186/s40478-019-0672-z

**Published:** 2019-02-12

**Authors:** Tomoko Miki, Osamu Yokota, Takashi Haraguchi, Takeshi Ikeuchi, Bin Zhu, Shintaro Takenoshita, Seishi Terada, Norihito Yamada

**Affiliations:** 1 Department of Neuropsychiatry, Okayama University Graduate School of Medicine, Dentistry and Pharmaceutical Sciences, 2-5-1 Shikata-cho, Okayama, 700-8558 Japan; 2 Department of Psychiatry, Kinoko Espoir Hospital, Okayama, Japan; 3 Department of Neurology, National Hospital Organization Minami-Okayama Medical Center, Okayama, Japan; 4 Department of Molecular Neuroscience, Brain Research Institute, Niigata University, Niigata, Japan

**Keywords:** Alzheimer’s disease, Cotton wool plaques, Disease duration, Presenilin 1 mutation, Spastic paraparesis

To the editor,

Presenilin 1 gene (*PSEN1*)-linked Alzheimer’s disease with cotton wool plaques (CWP-AD) is a rare variant that is often clinically characterized by dementia with spastic paraparesis [4]. CWPs are eosinophilic, round, large, non-cored, and Aβ-positive plaques with minor dystrophic neurites. The clinical heterogeneity of this variant remains unclear. Here, we describe an autopsy case of CWP-AD with a novel *PSEN1* mutation that showed slowly progressive cognitive and motor disturbances from the mid-20s with a very long disease duration of about 30 years.

The proband was a right-handed Japanese woman who initially presented with a decrease of calculation ability at age 25. Her brother initially exhibited slurred speech and bradykinesia at 39 years. Subsequently, parkinsonism, alien hand sign, and dementia developed. He died at 48 years. The proband’s mother, uncle, and maternal grandfather showed dementia and/or gait disturbance, and all died at 48 years. None of these relatives was genetically examined.

The proband exhibited muscle weakness of both hands and forgetfulness at 26 years. Clumsiness in the right upper and lower extremities developed at 30 years. She first went to the department of neurology at a general hospital and was suspected as having AD with parkinsonism. Dysarthria and dysphagia emerged at 34 and 35 years, respectively. Eleven years after the onset, she was admitted to the department of neurology at a university hospital. Neurological examination revealed limitation of upward and lateral gaze, bilaterally increased tendon reflex in all four extremities, bilaterally positive Babinski sign, spastic paraparesis, akinesia, and rigidity of the neck and four extremities. Parkinsonism was unresponsive to L-dopa treatment. She scored 20/30 points on the Mini-Mental State Examination [5], and on the WAIS-Revised, she obtained a verbal IQ score of 69, performance IQ score of 46, and full-scale IQ score of 54. Baseline blood and cerebrospinal fluid examinations were normal. She could walk without support until 37 years old. Brain MRI at 40 years demonstrated diffuse cerebral atrophy (Fig. 1a-d). At 42 years, she needed tube feeding due to dysphagia. ^99m^Tc-ECD single-photon emission computed tomography (SPECT) at 44 years disclosed hypoperfusion in the posterior part of the cingulate gyrus, precuneus, and parieto-occipital cortices (Fig. 2). Brain MRI at 54 years showed cerebral atrophy with severe dilatation of the ventricles (Fig. 1e and f). She died of respiratory failure at age 54 after a disease duration of 29 years. No respiratory support was given throughout the course. Her final neurological diagnosis was unclassifiable dementia.

**Fig. 1:**
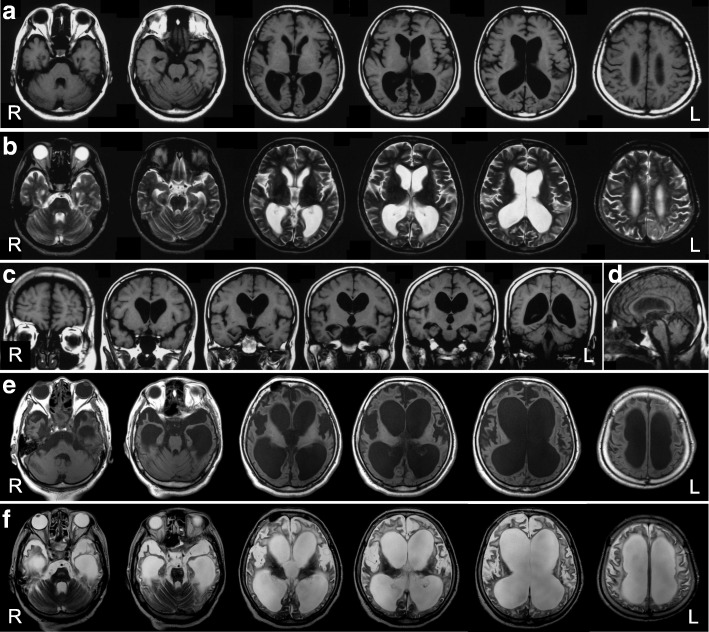
MR images of the present case. **a** T1-weighted horizontal, **b** T2-weighted horizontal, **c** T1-weighted coronal, and **d** T1-weighted sagittal images at 40 years. Evident bilateral atrophy of the hippocampus and symmetric white matter atrophy with occipital predominance are noted. The width of the corpus callosum is reduced (**c**). The brain stem doesn’t show evident atrophy (**d**). **e** T1-weighted horizontal and **f** T2-weighted horizontal images at 54 years. Diffuse cortical atrophy is progressed. The symmetric dilatation of the lateral ventricles becomes evident, suggesting the potential complication of idiopathic normal pressure hydrocephalus in addition to the atrophy of the white matter

**Fig. 2:**
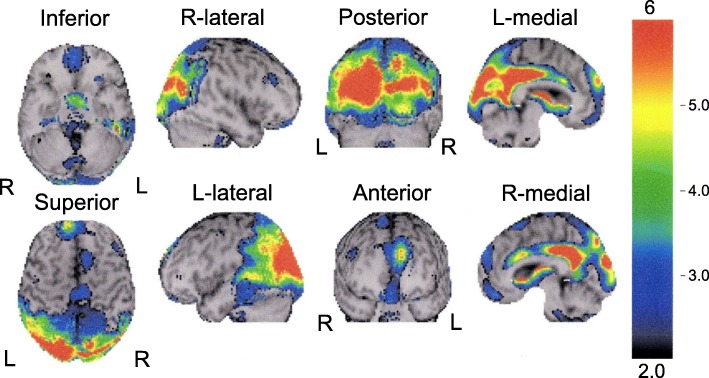
^99m^Tc-ECD SPECT images at age 44. The cerebral blood flow in the bilateral posterior cingulate gyri, precuneus, and parietal and occipital cortices is reduced with left-side predominance

The brain weighed 895 g before fixation. Macroscopically, severe atrophy in the neocortex (Fig. 3a-c) and marked depigmentation in the substantia nigra (Fig. 3d) and locus coeruleus (Fig. 3e) were noted. The pyramidal tract at the level of the medulla oblongata was atrophic (Fig. 3f). Histopathologically, abundant CWPs were noted throughout the cerebral cortex (Fig. 4a-g, Table 1). Neuritic plaques with dense amyloid cores were hardly noted in any region. Abundant Aβ deposits were noted in the cerebellum (Figs. 4h, 5c, and d) and spinal gray matter (Fig. 6e and f). Aβ42 rather than Aβ40 was predominantly accumulated in CWPs and cerebellar Aβ plaques (Fig. 5a-d). Remarkable cerebral amyloid angiopathy was also noted, although it was hardly related to CWPs spatially (Figs. 4c, g, and 5). The distributions of Aβ deposits and neurofibrillary changes were classified as Thal phase 5 [24] and Braak stage VI [1]. Viewing Congo red-stained sections with polarized light did not demonstrate apple green birefringence in CWPs (Fig. 7a and b). Neuronal loss associated with the proliferation of GFAP-positive astrocytes and Iba1-positive microglias was remarkable in the cerebral cortex and basal ganglia (Figs. 4a, 7c, d, and Table 1). Loss of Betz cells in the motor cortex (Fig. 6a) and degeneration of the pyramidal tract (Fig. 6b-d) were evident. Motor neurons in the spinal anterior horns and hypoglossal nuclei were spared in number (Fig. 6d). α-Synuclein-positive Lewy bodies were extensively distributed, corresponding to diffuse neocortical type Lewy body disease [13] and Braak Parkinson’s disease stage 5 [2]. Pigmented neurons in the substantia nigra were severely reduced in number (Fig. 8a-d). TDP-43-positive neurocytoplasmic inclusions, intranuclear inclusions, and short neurites were noted in the limbic region and temporal cortex, corresponding to Josephs stage III (Fig. 8e-g) [9]. No argyrophilic grain, tufted astrocyte, astrocytic plaque, FUS pathology, p62-positive inclusion in the cerebellar dentate nucleus, 1C2-positive inclusion, or pathological 3F4-positive lesion was noted.

**Fig. 3:**
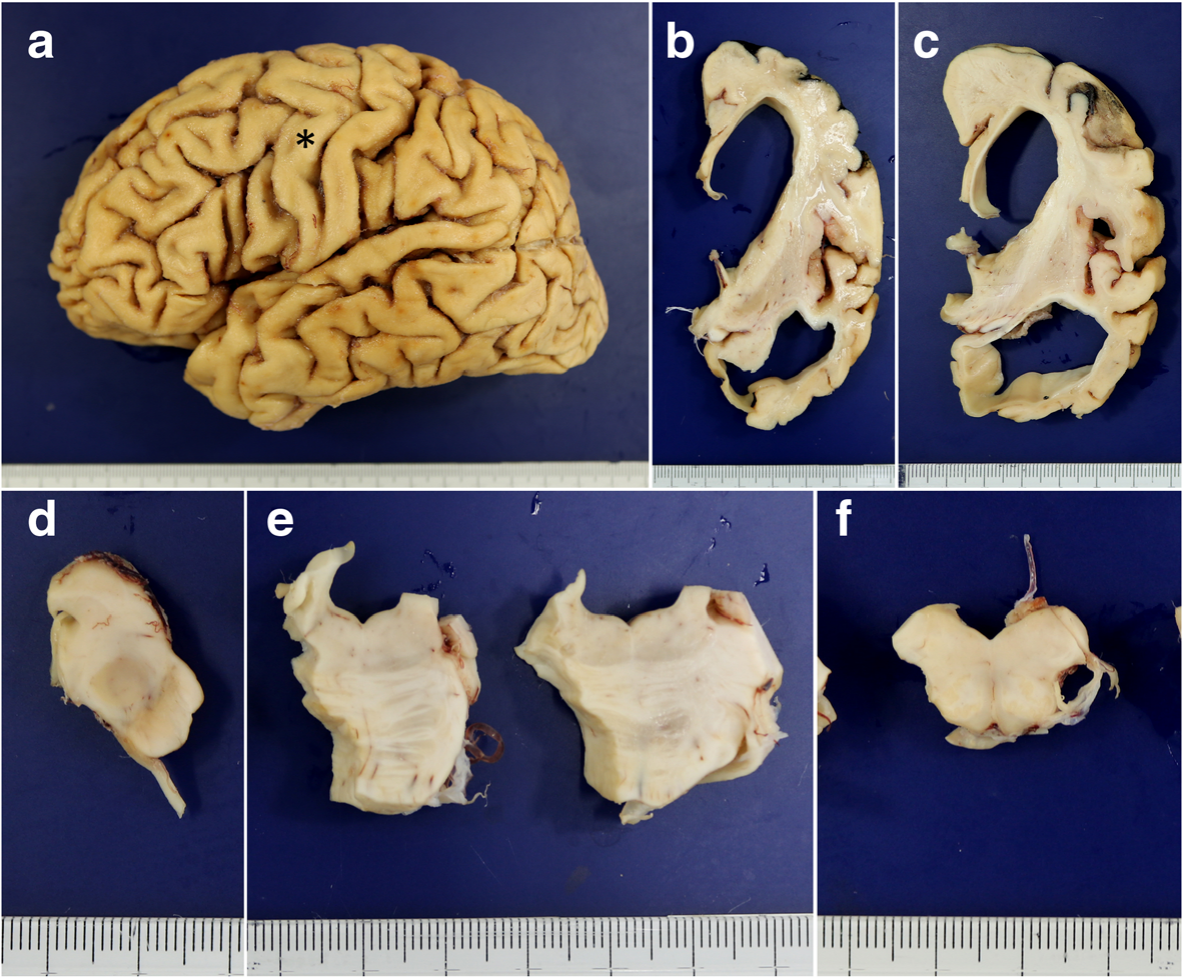
Macroscopic findings of the present case. **a** Lateral view of the left hemisphere. Severe diffuse atrophy including the precentral gyrus (an asterisk) is seen. **b** On a coronal section, severe atrophy is evident in the cortex and white matter of the frontal and temporal lobes. The basal ganglia also show severe atrophy. The width of the corpus callosum is severely reduced. **c** Remarkable atrophy of the hippocampus and parahippocampal gyrus. **d** Severe depigmentation in the substantia nigra. **e** Depigmentation in the locus coeruleus. **f** Severe atrophy in the pyramidal tract at the level of the medulla oblongata

**Fig. 4:**
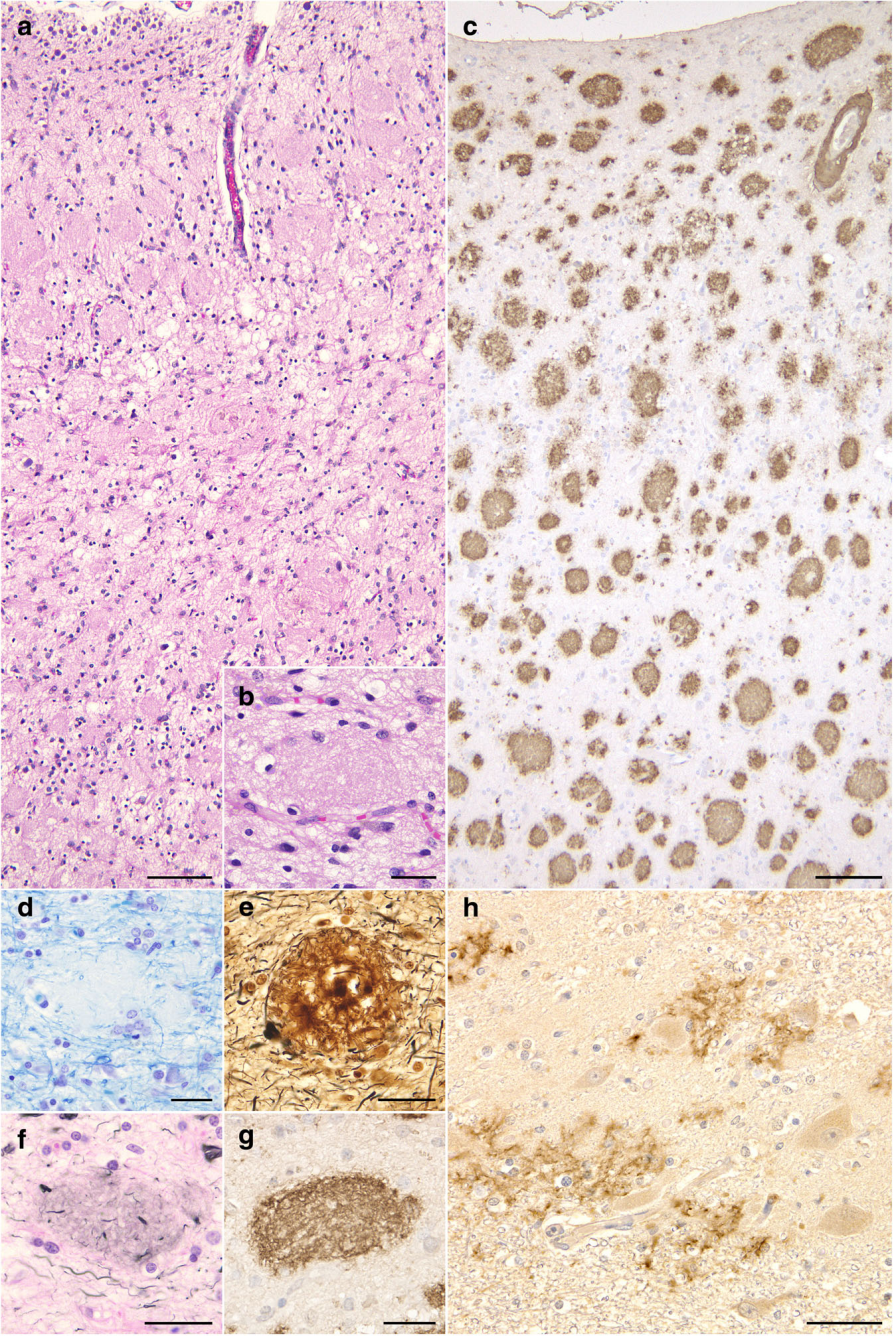
Histopathological findings of the present case. **a**, **b** Numerous eosinophilic, round, non-cored, and large cotton wool plaques in the insula cortex. The diameter of the plaques is often 100 μm or over. The rarefaction in the neuropil is also remarkable. Hematoxylin-eosin stain. **c** Aβ-positive abundant CWPs in all cortical layers. The inferior frontal gyrus. 12B2 immunohistochemistry. **d** A CWP showing a mass effect on around myelinated fibers in the neuropil. The inferior frontal gyrus. Klüver-Barrera stain. **e** Modified Bielschowsky silver stain showed heterogeneous argyrophilia but no clear amyloid core in a CWP. The middle frontal gyrus. **f** Gallyas silver stain shows only weak argyrophilia of homogeneous material composing a CWP. The inferior frontal gyrus. **g** A CWP strongly stained with an anti-Aβ antibody. The inferior frontal gyrus. 12B2 immunohistochemistry. **h** Aβ deposits in the dentate nucleus in the cerebellum. 12B2 immunohistochemistry. Scale bars = **a** 100 μm, **b** 30 μm, **c** 100 μm, **d**, **e**, **f**, **g** 30 μm, **h** 40 μm

**Table 1:** Distribution of lesions in the present case

	Neuronal loss/gliosis	Aβ deposits/CWPs	NFTs	Lewy bodies/Lewy neurites	TDP-43 positive NCIs/short neurites
Primary motor cortex	+++	+++++/+++	++++	−/−	−/−
Superior frontal gyrus	+++	+++++/+++++	++++	−/−	−/−
Middle frontal gyrus	+++	+++++/++++	++++	−/−	−/−
Inferior frontal gyrus	+++	+++++/++++	++++	−/−	−/−
Frontal white matter	+++	++/−	–	−/−	−/−
Superior temporal gyrus	+++	+++++/+++++	++++	−/−	−/−
Middle temporal gyrus	+++	+++++/+++++	++++	−/−	−/−
Inferior temporal gyrus	+++	+++++/+++++	++++	++/++	+/+
Lateral occipitotemporal gyrus	+++	+++++/+++++	++++	+/++	++/+
Temporal white matter	+++	++++/−	–	−/−	−/−
Parietal cortex	+++	+++++/++++	++++	−/−	−/−
Parietal white matter	+++	++++/−	–	−/−	−/−
Occipital cortex	+++	++++/+++	++++	−/−	−/−
Occipital white matter	+++	++++/−	–	−/−	−/−
Entorhinal cortex	+++	+++++/++++	++++	+++/++++	++++/−
Hippocampal CA1	+++	+++++/++++	++++	+/−	++++/−
Amygdala	+	+++++/++++	+++++	++++/++++	++++/++
Caudate nucleus	+++	+++++/+++++	++++	−/−	−/−
Putamen	+++	+++++/+++++	+++++	−/−	−/−
Globus pallidus	+++	++/−	++++	−/−	−/−
Subthalamic nucleus	n.a.	n.a.	n.a.	n.a.	n.a.
Oculomotor nucleus	–	−/−	++	−/−	−/−
Substantia nigra	+++	+/−	++++	−/+	−/−
Frontopontine tract	–	+/−	–	−/−	−/−
Corticospinal tract					
at level of cerebral peduncle	+++	−/−	–	−/−	−/−
at level of pons	+++	−/−	–	−/−	−/−
at level of medulla oblongata	+++	−/−	–	−/−	−/−
Locus coeruleus	++	+/−	++	+/−	−/−
Pontine nucleus	–	++/−	+++	−/−	−/−
Superior cerebellar peduncle	–	−/−	–	−/−	−/−
Transverse pontine fibers	–	−/−	–	−/−	−/−
Dorsal vagal nucleus	+	+/−	++	−/−	−/−
Hypoglossal nucleus	–	+/−	–	−/−	−/−
Inferior olivary nucleus	–	+/+	–	−/−	−/−
Cerebellum					
Molecular layer	+	++++/−	–	−/−	−/−
Purkinje cell layer	–	++/−	–	−/−	−/−
Granular layer	–	++++/−	–	−/−	−/−
Dentate nucleus	–	+++++/−	+++	−/−	−/−
White matter	++	+/−	–	−/−	−/−
Spinal cord					
Anterior horn	–	++/−	–	−/−	−/−
Corticospinal tract	+++	+/−	–	−/−	−/−

Neuronal loss -: none, +: mild, ++: moderate, +++: severe. The severity of the degeneration of the tract was evaluated with the following system using hematoxylin-eosin and Klüver-Barrera stains: - (no degeneration), neither loss of myelin nor glial proliferation was found; + (mild degeneration), slight myelin loss and gliosis without atrophy of the tract; ++ (moderate degeneration), evident myelin loss and gliosis with slight atrophy of the tract; +++ (severe degeneration), evident myelin loss and gliosis with severe atrophy of the tract. Aβ deposits and cotton wool plaques (CWPs) were semiquantitatively evaluated with the following staging system using 12B2 immunohistochemistry: -, no lesion; ±, one lesion in each anatomical region; +, one lesion per ×200 visual field; ++, 2 to 10 lesions per × 200 visual field; +++, 11 to 20 lesions per × 200 visual field; ++++, 21 to 50 lesions per × 200 visual field; +++++, 51 or more lesions per × 200 visual field. AT-8 positive neurofibrillary tangles (NFTs), Lewy body, Lewy neurites, TDP-43 positive neuronal cytoplasmic inclusions (NCIs), and TDP-43 positive short neurites were semiquantitatively evaluated with the following staging system using AT8, pSyn#64, and pS409/410–2 immunohistochemistry, respectively: -, no lesion; +, one lesion in the anatomical region; ++, two to four lesions in the anatomical lesion but less than one lesion per × 200 visual field; +++, one lesion per × 200 visual field; ++++, 2 to 10 lesions per × 200 visual field; +++++, over 11 lesions per × 200 visual field. n.a., not available

**Fig. 5:**
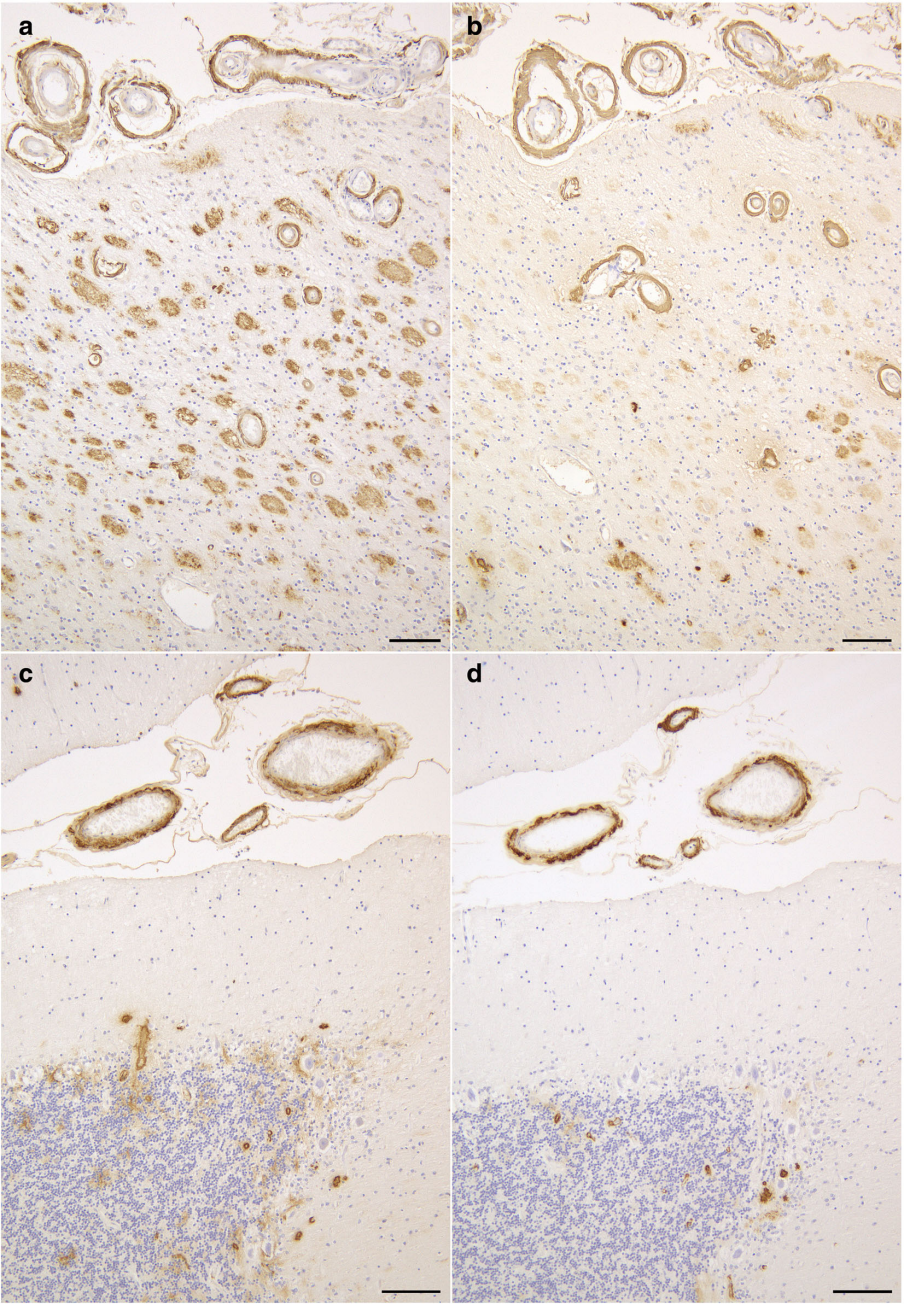
Aβ42 and Aβ40 immunohistochemistry on serial sections from the temporal cortex and cerebellar cortex. **a**, **b** Serial sections from the inferior temporal cortex. Aβ42 (**a**) rather than Aβ40 (**b**) is predominantly accumulated in CWPs as well as cerebral amyloid angiopathy. **c**, **d** Serial sections from the cerebellar cortex. Aβ42 (**c**) rather than Aβ40 (**d**) is predominantly accumulated in the cerebellar cortex, while both are almost equally accumulated in cerebral amyloid angiopathy. Scale bars = **a**, **b** 100 μm, **c**, **d** 30 μm

**Fig. 6:**
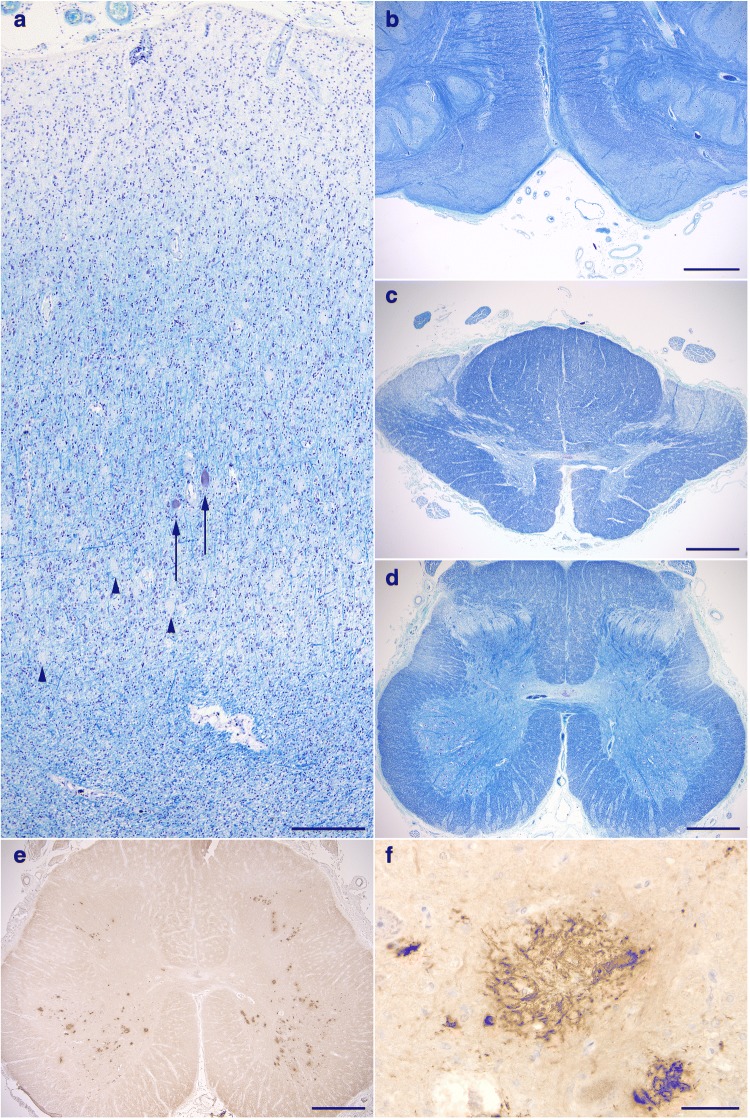
Degeneration of upper motor neurons. **a** Loss of Betz cells. (Arrows) A few remaining Betz cells. (Arrowheads) Numerous unstained CWPs that tend to be densely distributed in the deep cortical layers. **b** Degeneration with evident atrophy of the pyramidal tract at the level of the medulla oblongata. **c**, **d** Degeneration in the lateral tract in the thoracic (**c**) and lumbar cords (**d**). The anterior horn cells are well preserved in number (**d**). **e** Remarkable Aβ deposits in the anterior horns in the lumbar cord, while no Aβ deposit is seen in the corticospinal tract. **f** A high power view of Aβ deposits in the anterior horn in the lumbar cord. Scale bars = **a** 300 μm, **b-e** 1 mm, **f** 50 μm. **a-d** Klüver-Barrera stain. **e**, **f** 12B2 immunohistochemistry

**Fig. 7:**
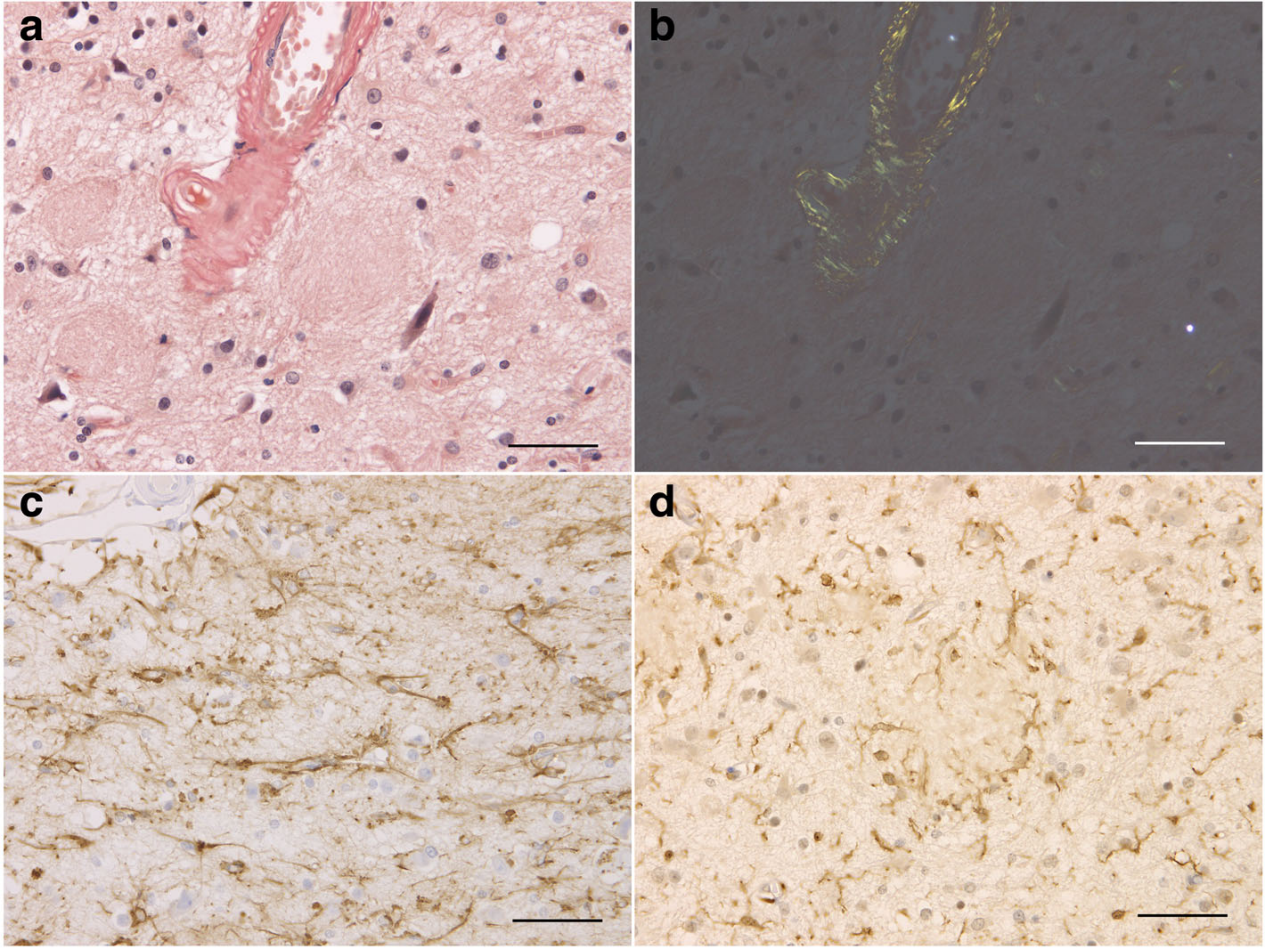
Absence of congophilia of CWPs with glial proliferation. **a**, **b** Cotton wool plaques (CWPs) on section stained with Congo red. Observation with polarized light demonstrates apple green birefringence in the amyloid angiopathy but not CWPs (**b**). The insular cortex. **c**, **d** GFAP-positive astrocytes (**c**) and Iba1-positive microglias (**d**) surrounding CWPs. The inferior temporal gyrus. All scale bars = 50 μm

**Fig. 8:**
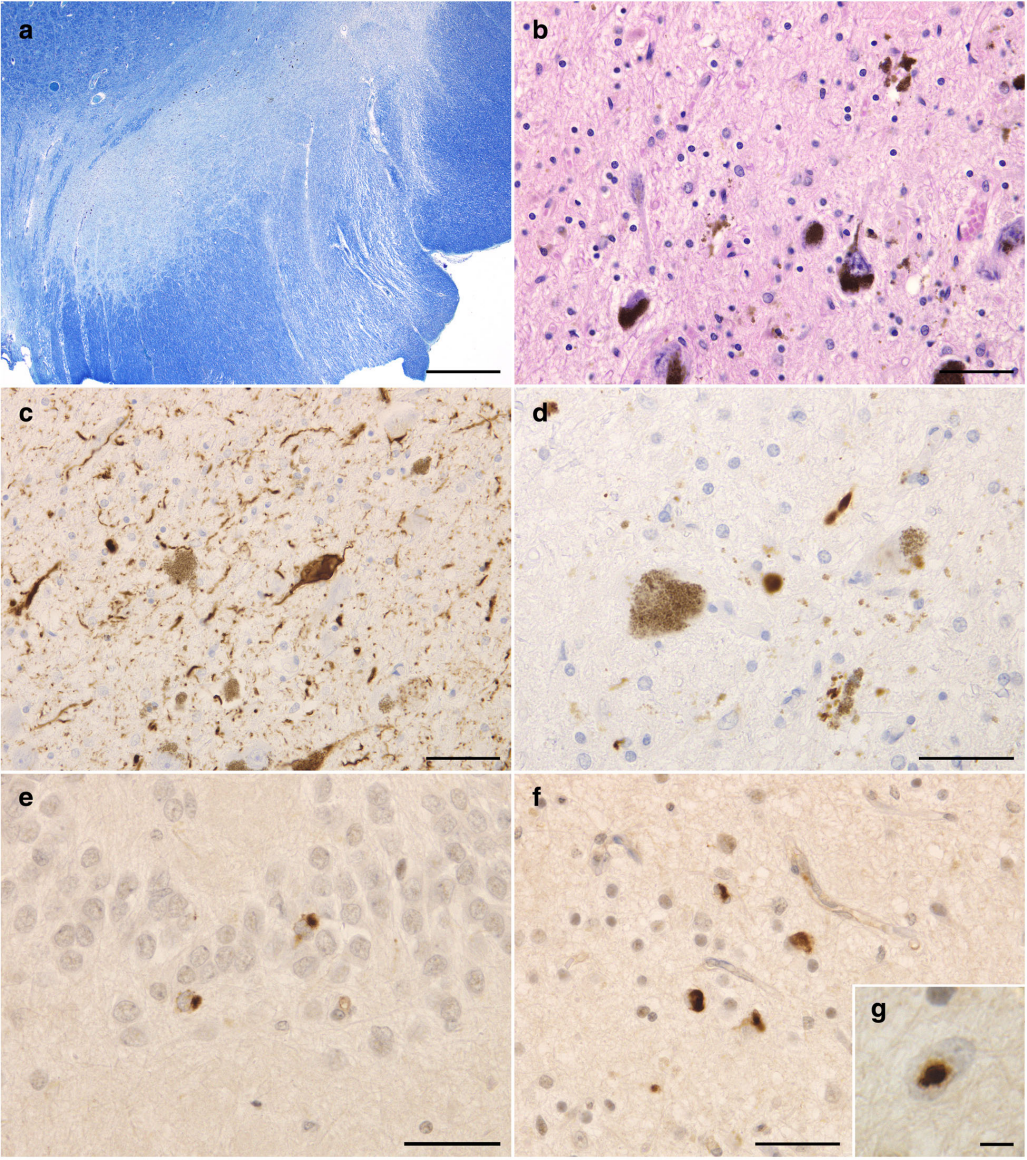
Pathological findings in the substantia nigra and limbic system. **a** Severe loss of pigmented neurons in the substantia nigra. The corticospinal tract is also severely degenerated. Klüver-Barrera stain. **b** Glial proliferation with free melanin in the substantia nigra. Hematoxylin-eosin stain. **c** Phosphorylated tau-positive dystrophic neurites and NFTs in the substantia nigra. AT8 immunohistochemistry. **d** Phosphorylated α-synuclein-positive Lewy neurites in the substantia nigra. Psyn#64 immunohistochemistry. (**e**, **f**) Phosphorylated TDP-43-positive neurocytoplasmic inclusions in the hippocampal dentate gyrus (**e**) and occipitotemporal gyrus (**f**). pS409/410–2 immunohistochemistry. **g** An intranuclear inclusion immunopositive for phosphorylated TDP-43. The occipitotemporal gyrus. pS409/410–2 immunohistochemistry. Scale bars = **a** 1 mm, **b**, **c** 50 μm, **d-f** 40 μm, **g** 5 μm

Mutational analysis of coding exons and flanking intronic sequences of *APP*, *PSEN1*, and *PSEN2* using frozen brain tissue demonstrated a novel c.1249G > A mutation (p.Gly417Ser) in exon 12 of *PSEN1* in the proband (Fig. 9a). The mutation is not present in the ExAC database (http://exac.broadinstitute.org) or jMorp database (https://jmorp.megabank.tohoku.ac.jp). There was no other mutation in *APP* or *PSEN2*. The *APOE* genotype was 3*4. A functional assay by establishing N2a cells that stably express the PS1 wild-type or p.G417S mutant [7] and sandwich ELISA demonstrated that the expression of *PSEN1* p.G417S resulted in increases in the Aβ42 and Aβ42/40 ratio, which were significantly higher than those in wild-type-expressing cells, suggesting that the mutation was likely to be causative in the present case (Fig. 9b. See details of methods in Additional files 1 and 2 [7]).

**Fig. 9:**
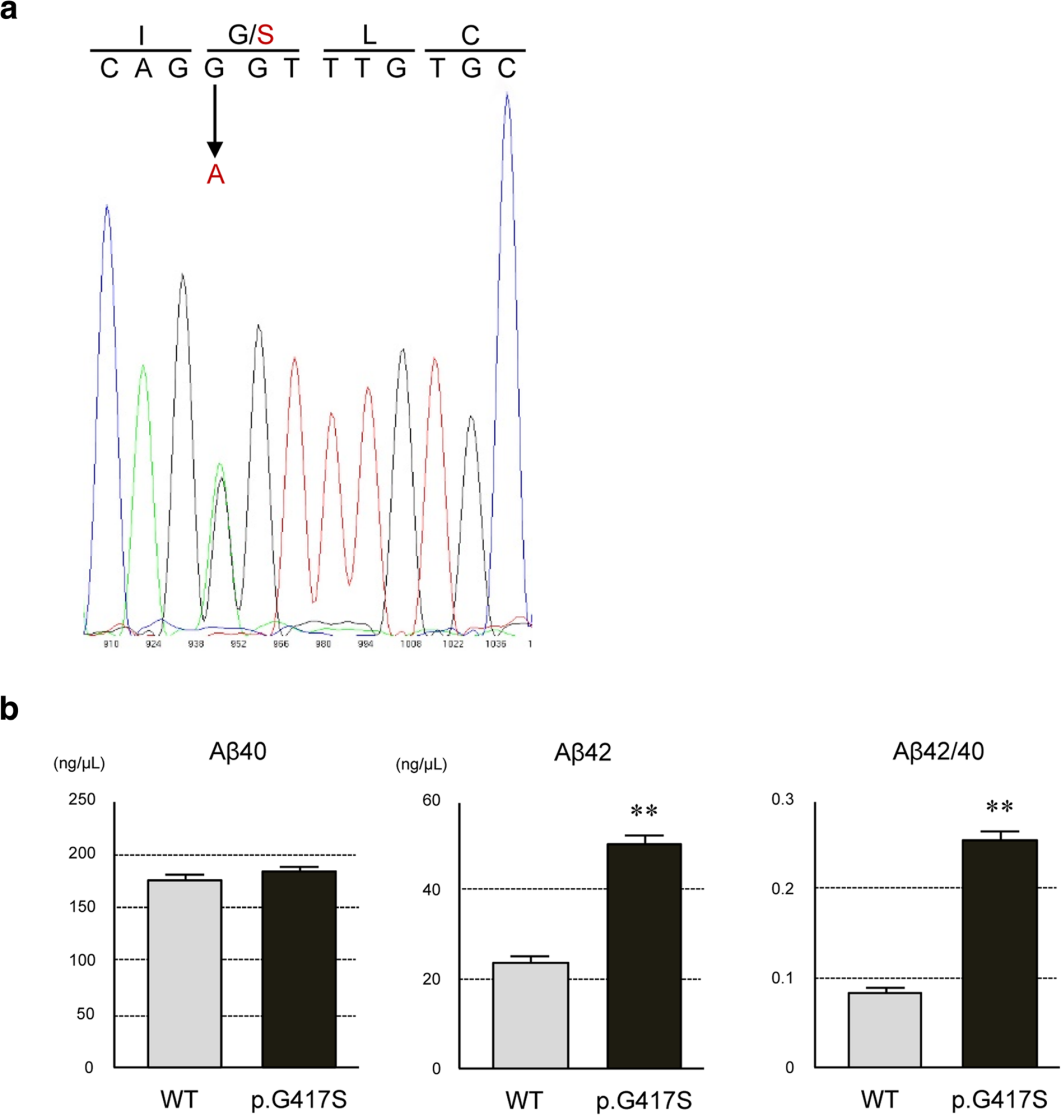
Detection of novel *PSEN1* mutation and functional assay. **a** Direct sequencing of *PSEN1* exon 12 of the patient demonstrated a novel mutation of c. 1249G > A indicated by arrow, resulted in a missense mutation of p.Gly417Ser. This mutation is predicted as probably damaging with a score of 0.979 by Polyphen-2 and a CADD score of 29.6. **b** The level of Aβ42 and the ratio of Aβ42/40 were significantly increased in the media of cells stably expressing mutant PS1 of p.G417S compared with those of wild-type. Data were plotted as mean ± SEM (*n* = 3). **P < 0.01

The most noteworthy clinical features in the present case were the young age at onset and very long disease duration. It was reported that the mean age at onset in 564 patients with *PSEN1*-linked AD was 43.3 ± 8.6 years, and that the interquartile range of the disease duration was five to 11 years [19]. In the previous 27 *PSEN1*-linked CWP-AD cases with sufficient clinical information that we reviewed (Table 2 [3, 4, 6, 8, 10, 12, 14, 15, 17, 18, 20–23]), the age at onset ranged from 29 to 58 years (mean: 45.4 ± 8.5 years), and the disease duration was from 3 to 20 years (mean: 9.9 ± 5.8 years) (Fig. 10a–c). Spastic paraparesis and parkinsonism were described in 15 (55.6%) and 6 cases (22.2%), respectively. As far as we know, the present case showed the youngest age at onset and the longest disease duration. In some CWP-AD cases, neurons surrounding CWPs tend to be spared in number and inflammatory changes are often relatively mild [4, 11, 25, 26]. However, our case showed remarkable neuronal loss with tissue rarefaction in the cerebral cortex. It may be explained by the extremely long disease duration. Interestingly, we recently found a poster abstract in which two siblings having dementia, spastic paraplegia, and the same *PSEN1* mutation was reported [16]. Although the information was limited, the ages at onset in these siblings were 32 and 36 years, respectively. To our knowledge, these individuals were not included in our pedigree. As shown in Fig. 10, the ages at onset in these clinical cases, like that in our case, are relatively young among previously reported CWP-AD cases. What factors besides mutations affect the age at onset and speeds of tissue degeneration and clinical progression in CWP-AD cases remain unclear. However, clinicians should be aware at least that the differential diagnosis of slowly progressive cognitive decline with spasticity and parkinsonism in young adults includes CWP-AD.

**Table 2:** Clinical and pathological features in the present case (case 1) and previously reported *PSEN1*-linked CWP-AD cases (cases 2–28)

Case	Sex	Age at onset (y)	Age at death (y)	Duration (y)	Initial symptoms	Spastic paraparesis	Parkinsonism	Brain weight (g)	CWPs	Lewy body disease	*PSEN1* mutation	References
1	f	25	54	29	cognitive impairment	+	+	895	+	diffuse	G417S	Present case
2	n.d.	29	n.d.	n.d.	spastic paraparesis	+	n.d.	n.d.	+	n.d.	P436Q	Houlden H et al. [6]
3	m	30s	48	n.a.	disorientation	–	n.d.	n.d.	+	n.d.	L420R	Shrimpton AE et al. (II:1) [20]
4	f	31	43	12	forgetfulness	–	+	1170	+	–	L420R	Niwa A et al. [14]
5	m	34	39	5	morbid jealousy	–	n.d.	1550	+	n.d	deletion of exon 9 sequence from *PSEN1* transcripts	Brooks WS et al. (EOFAD-2 IV:45) [3]
6	m	34	52	18	parkinsonism	–	+	1150	+	diffuse	in-frame 3bp ACC deletion in exon 12	Ishikawa A et al. [8]
7	n.d.	36	n.d.	n.d.	spastic paraparesis	+	n.d.	n.d.	+	n.d.	Δ83,84IM	Houlden H et al. [6]
8	f	37	50	13	cognitive dysfunction, parkinsonism	+	+	740	+	limbic	G217D	Takao M et al. (case III-2) [23]
9	f	40	biopsy	n.a.	cognitive impairment	+	n.d.	biopsy	+	n.d.	E280G	O’Riordan S et al. (patient 3) [17]
10	f	41	45	4	cognitive decline	–	n.d.	992	+	n.d.	G- > T exon 9 splice acceptor mutation	Brooks WS et al. (EOFAD-3 II:10) [3]
11	m	41	46	5	dementia	n.d.	n.d.	n.d.	+	n.d.	deletion of exon 9	Smith MJ et al. (case II:12) [22]
12	m	42	46	4	antiflexion gait	+	+	1150	+	–	G217D	Takao M et al. (case III-1) [23]
13	f	46	52	6	memory loss and disorientation	–	n.d.	1144	+	n.d.	G217R	Norton JB et al. (case 4:4) [15]
14	m	46	65	19	depression	+	+	1100	+	–	P264L	Martikinen P et al. (case 3) [12]
15	f	47	51	4	cognitive decline	+	n.d.	n.d.	+	n.d.	G- > T exon 9 splice acceptor mutation	Brooks WS et al. (EOFAD-3 II:8) [3]
16	m	47	67	20	spasticity and weakness in legs	+	n.d.	1110	+	–	E280G	Sinha N et al. [21]
17	f	50	53	3	spastic paraparesis	+	n.d.	n.d.	+	n.d.	deletion of exon 9	Smith MJ et al. (case III:9) [22]
18	f	50	60	10	cognitive decline	–	n.d.	918	+	n.d.	deletion of exon 9 sequence from *PSEN1* transcripts	Brooks WS et al. (EOFAD-2 III:18) [3]
19	f	51	68	17	memory impairment	–	+	1050	+	–	P264L	Martikinen P et al. (case 1) [12]
20	m	52	56	4	cognitive decline	–	n.d.	910	+	n.d.	deletion of exon 9 sequence from *PSEN1* transcripts	Brooks WS et al. (EOFAD-2 IV:23) [3]
21	m	52	56	4	dementia	n.d.	n.d.	n.d.	+	n.d.	deletion of exon 9	Smith MJ et al. (case II:7) [22]
22	m	52	67	15	memory difficulty and weakness in both legs	+	n.d.	890	+	n.d.	E280Q	Rogaeva E et al. [18]
23	f	54	63	9	dementia and spastic paraparesis	+	n.d.	n.d.	+	n.d.	deletion of exon 9	Smith MJ et al. case (III:7) [22]
24	m	54	64	10	dementia	+	n.d.	1360	+	n.d.	deletion of exon 9	Crook R et al. (case III:15) [4]
25	m	55	61	6	back pain, stiffness of legs	+	n.d.	n.d.	+	n.d.	deletion of exon 9 splice site	Crook R et al. (patient III:9) [4]
26	m	57	69	12	dementia	+	n.d.	1075	+	n.d.	deletion of exon 9	Crook R et al. (case III:14) [4]
27	m	58	75	17	memory impairment	+	–	1320	+	–	P264L	Martikinen P et al. (case 2) [12]
28	f	n.d.	60	n.a.	n.d.	–	n.d.	n.d.	+	n.d.	L271V	Kwok JB et al. (III:28) [10]

Cases are shown in the order of the age at onset. m, male; f, female; +, present; −, absent; n.d., not described; n.a., not available; CWPs, cotton wool plaques; diffuse, diffuse neocortical type Lewy body disease [13]; limbic, limbic type Lewy body disease [13]

**Fig. 10:**
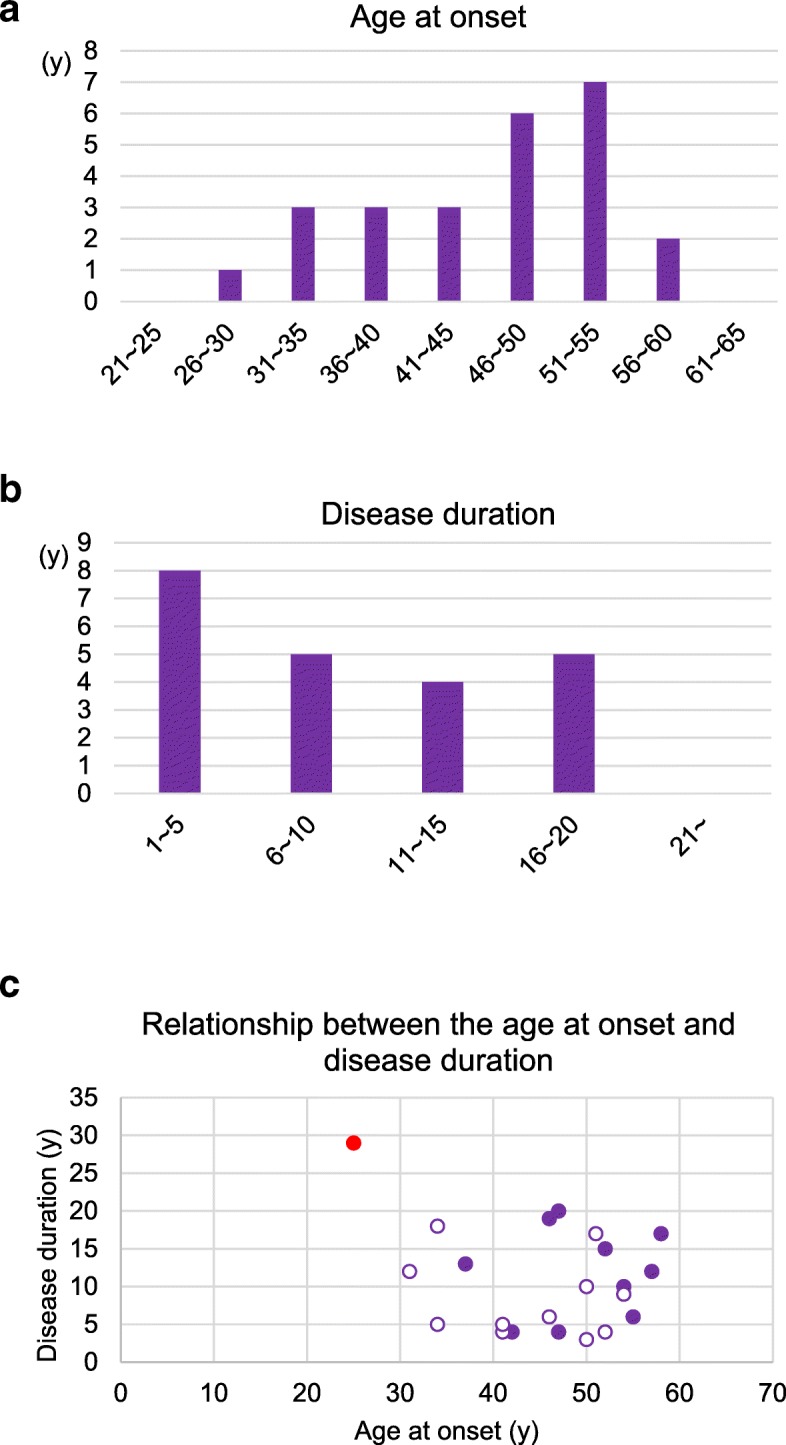
Distribution of the age at onset and disease duration in previously reported CWP-AD cases. **a** The age at onset in previously reported CWP-AD cases due to *PSEN1* mutations. **b** The disease duration in previously reported CWP-AD cases due to *PSEN1* mutations. **c** The relationship between the age at onset and disease duration in the present case and previously reported CWP-AD. The data of cases 1 to 28 were extracted from the references cited in Table 2. No significant correlation between the age at onset and disease duration was demonstrated by Spearman rank order correlation analysis when all cases whose disease duration was available were examined (*ρ* = − 0.101, *p* = 0.65) or when only previous cases were examined (*ρ* = 0.058, *p* = 0.80). These findings suggest that young age at onset is not necessarily a factor that predicts rapid progression or short disease duration in CWP-AD cases. Red solid circle: the present case, purple solid circle: previously reported CWP-AD cases with spastic paraparesis, purple open circle: previously reported CWP-AD cases without spastic paraparesis

## Additional files

Additional file 1: Details of methods. (DOCX 20 kb)

Additional file 2: Antibodies used in this study. (DOCX 24 kb)

## Abbreviations

AD: Alzheimer’s disease
CWP: Cotton wool plaque
CWP-AD: Alzheimer’s disease with cotton wool plaques
*PSEN1*: Presenilin 1 gene
