# Supplementary material for: Young adult-onset, very slowly progressive cognitive decline with spastic paraparesis in Alzheimer’s disease with cotton wool plaques due to a novel presenilin1 G417S mutation
Source: Acta Neuropathol Commun. 2019 Feb 12;7:19. doi: 10.1186/s40478-019-0672-z (PMC6371429; doi:10.1186/s40478-019-0672-z)
Supplement: Supplementary file 1 — Details of methods. (DOCX 20 kb) [file 40478_2019_672_MOESM1_ESM.docx]

**Additional file 1**

**Young adult-onset, very slowly progressive cognitive decline with spastic paraparesis in Alzheimer’s disease with cotton wool plaques due to a novel presenilin1 G417S mutation**

Tomoko Miki^1,2^, Osamu Yokota^1,2,3^, Takashi Haraguchi^3^, Takeshi Ikeuchi^4^, Bin Zhu^4^, Shintaro Takenoshita^1^, Seishi Terada^1^, Norihito Yamada^1^

**Conventional neuropathological examination**

A standardized neuropathological evaluation was done. Brain tissue samples were fixed post mortem with 10% formaldehyde and embedded in paraffin. Ten-µm-thick sections from the frontal, temporal, parietal, occipital, insular, and cingulate cortices, hippocampus, amygdala, basal ganglia, midbrain, pons, medulla oblongata, cerebellum, and spinal cord were prepared. For the standardized neuropathological assessment, sections were stained with hematoxylin-eosin and Klüver-Barrera stains, and selected regions with modified Bielschowsky silver, Gallyas methods, and Congo red.

**Immunohistochemistry**

Formalin-fixed paraffin sections were cut at 6 μm thickness and included the frontal, temporal, parietal, and occipital cortices, basal ganglia, brain stem, cerebellum, and spinal cord for the standard evaluation. Deparaffinized sections were incubated with 1% H_2_O_2_ in methanol for 30 min to eliminate endogenous peroxidase activity, and washed in phosphate-buffered saline (PBS, pH 7.4). After blocking with 10% normal serum, sections were incubated overnight at 4°C with primary antibodies (Additional file 2). When using anti-tau, anti-α-synuclein, and anti-TDP-43 antibodies and MAB1510, p62-N, p62-C, SMI31, GFAP, CD68, Iba1, and 3F4, sections were autoclaved for 10 min in 10 mM sodium citrate buffer at 121°C for antigen retrieval. When using anti-Aβ antibodies, anti-4R tau, RD4, RD3, T22, and 1C2, sections were autoclaved for 10 min in 10 mM sodium citrate buffer at 121°C and treated with 70% formic acid for 10 min. After three 5-min washes in PBS, sections were incubated in biotinylated secondary antibody for 30 min, and then in avidin-biotinylated horseradish peroxidase complex (ABC Elite kit, Vector, Burlingame, CA, USA) for 60 min. After three 5-min washes in PBS, the peroxidase labeling was visualized with 0.2% 3,3’-diaminobenzidine (DAB) as the chromogen. Sections were lightly counterstained with hematoxylin.

**Mutational analysis**

Genomic DNA was extracted from frozen brain tissue of the patient. Mutational analysis was performed using sequences of both strands of PCR-amplified coding exons and flanking intronic sequences of *APP*, *PSEN1*, and *PSEN2* as reported previously [1]. The *APOE* genotype was determined by a Taq-Man based assay. We conducted *in silico* analysis using the PolyPhen-2 and CADD algorithms to predict the pathogenicity of a novel mutation.

**Cell culture and ELISA**

The cDNA encoding the PS1 mutant of p.G417S was generated using PCR-based mutagenesis kit (Invitrogen). Neuro2a (N2a) cells stably expressing wild-type or mutant PS1 were established as reported previously [1]. Conditioned media were collected to analyze the levels of amyloid-β (Aβ) species. Endogenous levels of Aβ40 and Aβ42 were quantified by sandwich ELISA (Wako) as reported previously [1].

**References**

1. Ikeuchi T, Kaneko H, Miyashita A, Nozaki H, Kasuga K, Tsukie T et al (2008) Mutational analysis in early-onset familial dementia in the Japanese population. The role of PSEN1 and MAPT R406W mutations. Dement Geriatr Cogn Disord 26:43-49.
