# Supplementary material for: Young adult-onset, very slowly progressive cognitive decline with spastic paraparesis in Alzheimer’s disease with cotton wool plaques due to a novel presenilin1 G417S mutation
Source: Acta Neuropathol Commun. 2019 Feb 12;7:19. doi: 10.1186/s40478-019-0672-z (PMC6371429; doi:10.1186/s40478-019-0672-z)
Supplement: Supplementary file 2 — Antibodies used in this study. (DOCX 24 kb) [file 40478_2019_672_MOESM2_ESM.docx]

**Additional file 2**

**Young adult-onset, very slowly progressive cognitive decline with spastic paraparesis in Alzheimer’s disease with cotton wool plaques due to a novel presenilin1 G417S mutation**

Tomoko Miki^1,2^, Osamu Yokota^1,2,3^, Takashi Haraguchi^3^, Takeshi Ikeuchi^4^, Bin Zhu^4^, Shintaro Takenoshita^1^, Seishi Terada^1^, Norihito Yamada^1^

**Antibodies used in this study**

Antibody Species/type Dilution Epitope Source

AT8 Mouse/monoclonal 1:1,000 Tau phosphorylated at Ser 202 Innogenetics

AT100 Mouse/monoclonal 1:500 Tau phosphorylated at Ser 212 and Thr 214 Innogenetics

AT180 Mouse/monoclonal 1:500 Tau phosphorylated at Thr 231 Innogenetics

AT270 Mouse/monoclonal 1:500 Tau phosphorylated at Thr 181 Innogenetics

PHF-1 Mouse/monoclonal 1:250 Tau phosphorylated at Ser 396 and Ser 404 [3]

Alz-50 Mouse/monoclonal 1:250 Tau epitope at aa 5-15 [1]

MC-1 Mouse/monoclonal 1:250 Conformation-dependent tau epitope [4]

within aa 312-322

T46 Mouse/monoclonal 1:500 Phosphorylation independent epitope Invitrogen

in aa 404-441 of human tau

T22 Rabbit/polyclonal 1:500 Tau oligomer [5]

RD3 Mouse/monoclonal 1:2,000 3 repeat tau-specific anti-tau antibody Millipore [2]

RD4 Mouse/monoclonal 1:100 4 repeat tau-specific anti-tau antibody Millipore [2]

Anti-4R tau Rabbit/polyclonal 1 :2,000 4 repeat tau-specific anti-tau antibody Cosmo Bio

MAB1510 Mouse/monoclonal 1:500 Ubiquitin Millipore

p62-N Guinea pig/polyclonal 1:200 N-terminus of p62 protein Progen Biotechnik

p62-C Guinea pig/polyclonal 1:500 C-terminus of p62 protein Progen Biotechnik

12B2 Mouse/monoclonal 1:100 Aβ(11–28) IBL

4G8 Mouse/monoclonal 1:50 Aβ(17–24) Covance

6E10 Mouse/monoclonal 1:50 Aβ(1–16) Covance

FCA3340 Rabbit/polyclonal 1:100 Aβ(1–40) Millipore

FCA3542 Rabbit/polyclonal 1:100 Aβ(1–42) Millipore

pSyn#64 Mouse/monoclonal 1:5,000 Phosphorylated α-synuclein Wako

pS409/410-2 Rabbit/polyclonal 1:5,000 Phosphorylated TDP-43 Cosmo Bio

HPA008784 Rabbit/polyclonal 1:200 FUS Sigma-Aldrich

SMI31 Mouse/monoclonal 1:500 Phosphorylated neurofilament Sternberger

GFAP Rabbit/polyclonal 1:100 Glial fibrillary acidic protein Dako

CD68 Mouse/monoclonal 1:500 CD68 Dako

Iba1 Rabbit/polyclonal 1:1000 C-terminus of Iba1 Wako

1C2 Mouse/monoclonal 1:10,000 Polyglutamine Millipore

3F4 Mouse/monoclonal 1:1,000 Prion protein Covance

**References**

1. Bowser R, Giambrone A, Davies P (1995) FAC1, a novel gene identified with the monoclonal antibody Alz50, is developmentally regulated in human brain. Dev Neurosci 17:20-37.
2. de Silva R, Lashley T, Gibb G, Hanger D, Hope A, Reid A, et al. (2003) Pathological inclusion bodies in tauopathies contain distinct complements of tau with three or four microtubule-binding repeat domains as demonstrated by new specific monoclonal antibodies. Neuropathol Appl Neurobiol 29: 288-302.
3. Greenberg SG, Davies P (1990) A preparation of Alzheimer paired helical filaments that displays distinct tau proteins by polyacrylamide gel electrophoresis. Proc Natl Acad Sci USA 87:5827-5831.
4. Jicha GA, Bowser R, Kazam IG, Davies P (1997) Alz-50 and MC-1, a new monoclonal antibody raised to paired helical filaments, recognize conformational epitopes on recombinant tau. J Neurosci Res 48:128-132.
5. Lasagna-Reeves CA, Castillo-Carranza DL, Sengupta U, Sarmiento J, Troncoso J, Jackson GR, et al. (2012) Identification of oligomers at early stages of tau aggregation in Alzheimer's disease. FASEB J. 26:1946-1959.
